# Fiber-Coupled Quartz-Enhanced Photoacoustic Spectroscopy System for Methane and Ethane Monitoring in the Near-Infrared Spectral Range

**DOI:** 10.3390/molecules25235607

**Published:** 2020-11-28

**Authors:** Giansergio Menduni, Fabrizio Sgobba, Stefano Dello Russo, Ada Cristina Ranieri, Angelo Sampaolo, Pietro Patimisco, Marilena Giglio, Vittorio M.N. Passaro, Sebastian Csutak, Dario Assante, Ezio Ranieri, Eric Geoffrion, Vincenzo Spagnolo

**Affiliations:** 1 PolySense Lab, Dipartimento Interateneo di Fisica, University and Politecnico of Bari, CNR-IFN, Via Amendola 173, 70126 Bari, Italy; giansergio.menduni@poliba.it (G.M.); fabrizio.sgobba@uniba.it (F.S.); stefano.dellorusso@uniba.it (S.D.R.); cristinaranieri4@gmail.com (A.C.R.); angelo.sampaolo@poliba.it (A.S.); pietro.patimisco@uniba.it (P.P.); marilena.giglio@uniba.it (M.G.); 2 Photonics Research Group, Dipartimento di Ingegneria Elettrica e dell’Informazione, Politecnico di Bari, Via Orabona 4, 70126 Bari, Italy; vittorio.passaro@poliba.it; 3 Faculty of Engineering, Uninettuno University, 00186 Rome, Italy; d.assante@uninettunouniversity.net; 4 Independent Consultant, 16300 Park Row Dr, Houston, TX 77084, USA; csutak@gmail.com; 5 Dipartimento di Biologia, Università degli Studi di Bari, Via Orabona 4, 70126 Bari, Italy; ezio.ranieri@uniba.it; 6 Thorlabs Canada ULC, 361 Boulevard Montpellier, Saint-Laurent, QC H4N 2G6, Canada; EGeoffrion@thorlabs.com

**Keywords:** quartz enhanced photoacoustic spectroscopy, near-IR fiber combiner, methane, ethane

## Abstract

We report on a fiber-coupled, quartz-enhanced photoacoustic spectroscopy (QEPAS) near-IR sensor for sequential detection of methane (CH_4_ or C1) and ethane (C_2_H_6_ or C2) in air. With the aim of developing a lightweight, compact, low-power-consumption sensor suitable for unmanned aerial vehicles (UAVs)-empowered environmental monitoring, an all-fiber configuration was designed and realized. Two laser diodes emitting at 1653.7 nm and 1684 nm for CH_4_ and C_2_H_6_ detection, respectively, were fiber-combined and fiber-coupled to the collimator port of the acoustic detection module. No cross talk between methane and ethane QEPAS signal was observed, and the related peak signals were well resolved. The QEPAS sensor was calibrated using gas samples generated from certified concentrations of 1% CH_4_ in N_2_ and 1% C_2_H_6_ in N_2_. At a lock-in integration time of 100 ms, minimum detection limits of 0.76 ppm and 34 ppm for methane and ethane were achieved, respectively. The relaxation rate of CH_4_ in standard air has been investigated considering the effects of H_2_O, N_2_ and O_2_ molecules. No influence on the CH_4_ QEPAS signal is expected when the water vapor concentration level present in air varies in the range 0.6–3%.

## 1. Introduction

Leakages from oil and gas pipelines and wildland fires significantly contribute to the greenhouse effect and degrade the air quality. Methane (CH_4_ or C1) and ethane (C_2_H_6_ or C2) represent the most significant environmental markers for the identification and tracking of the abovementioned pollution sources [1,2]. During these events, C1 and C2 concentrations can reach up to a few percent and the C2/C1 concentration ratio can be used to characterize the pollution source [3,4,5,6,7,8]. Thereby, real-time and in-situ monitoring of CH_4_ and C_2_H_6_ to detect and track concentration gradients in air would allow a prompt spotting of early fires in vast areas or localization of leakage origins in pipelines. Unmanned aerial vehicles (UAVs) can complement satellite technology and better monitor pollution markers’ environmental concentrations because of their improved performances in terms of UAV swarm control/coordination and on-board machine learning [9]. The gas sensors to be mounted on UAVs must be compact, lightweight and rugged to improve their time of flight and power consumption. High sensitivity is also requested to recognize positive variations with respect to the average concentration in air and guide the UAVs to the localization of the pollution sources. Among the most sensitive optical techniques [10,11], quartz-enhanced photoacoustic spectroscopy (QEPAS) has proven to be one of the prominent technologies capable of addressing these application requirements with the added value of the sensor modularity. QEPAS does not require any optical detector, is immune to environmental noise and can operate in a wide range of temperatures and pressures [12]. Together with its proven reliability, these features represent the main distinct advantages as compared to other laser-based techniques for environmental monitoring and in-situ detection on UAVs.

QEPAS employs a quartz tuning fork (QTF) as a sharply resonant acoustic transducer to detect a weak photoacoustic excitation, with the advantage of using extremely small sampling volumes [12]. The QTF is positioned inside a gas cell, thereby isolating the target sample from the surrounding environment and allowing the gas pressure to be controlled. Modulated light, focused between the QTF prongs, generates an acoustic wave when the laser wavelength is resonant with the targeted gas absorption bands. The quartz piezoelectric properties allow the prong motion generated by the pressure wave to be transduced into an electric signal, which is then amplified by means of a transimpedance or voltage amplifier [13,14,15] and demodulated by a lock-in amplifier. The acquired signal is proportional to the target gas concentration [16,17]. All laser-based sensors’ ability to discriminate absorption features related to different gases in a mixture is due to the high spectral selectivity of the optical sources employed. Suppose the target gas species are represented by linear or very simple molecules. In that case, the absorption bands will consist of well separated lines that usually allow discriminating the absorption features from different components and obtaining independent calibration curves for each species. When broadband absorptions related to more structured molecules overlap each other, several algorithms based on multiple linear regressions, partial least squares or multivariate analysis can be implemented to quantify the concentration of each gas [18,19,20,21,22,23].

Due to their light weight, compactness and low power consumption, fiber-coupled near-IR laser diodes are the best choice in terms of optical sources to be employed in UAV-based sensing. The ease of implementation for these devices makes feasible sensor architectures in which many different diodes can be combined through solid core optical fibers. Multiple laser sources in a QEPAS sensor enable multiple gas detection and/or can extend the detection range for a single target component in a mixture.

To empower real-time environmental monitoring based on unmanned vehicles exploration, we report in this work a novel, lightweight, low-power-consumption, fiber-based QEPAS sensor devoted to methane and ethane detection in the near-IR spectral range. The sensor architecture exploited a custom laser beam combiner and an innovative acoustic detection module (ADM), mounting a fiber collimator port instead of an optical window as in standard ADMs. The output beams of two pigtailed laser diodes were initially fiber combined and then collimated through a spectrophone mounting a custom T-QTF [24], using a fiber collimator. In this way, any free-space optical system was avoided and for a future sensor implementation, no issues related to vibrations produced by mobile vehicles or drones were expected. With this configuration, a sequential C1–C2 detection scheme was implemented and sensitivities in the parts per million and parts per billion concentration scales were achieved for C2 and C1, respectively.

## 2. Fiber-Based, Quartz-Enhanced Photoacoustic Sensor Architecture

**Figure 1:**
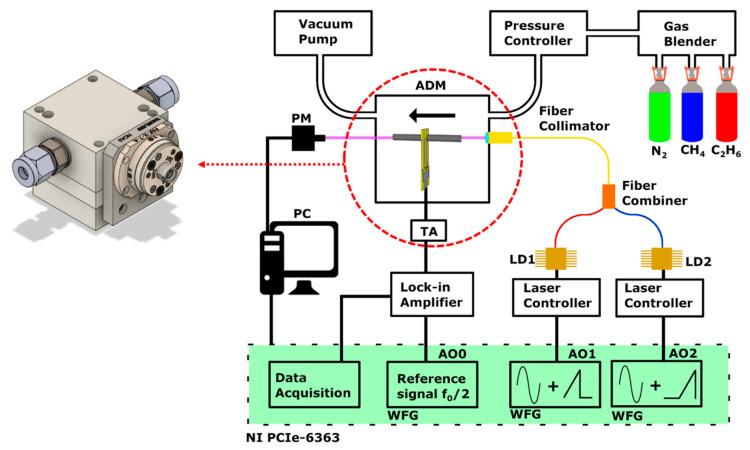
Schematic of the experimental apparatus: ADM—acoustic detection module, LD1—laser diode 1, LD2—laser diode 2, TA—transimpedance amplifier, PM—power meter, PC—personal computer, WFG—waveform generator. The black arrow indicates the gas flow through the ADM. A detailed design of the fiber-coupled ADM is also shown.

The architecture of the fiber-based QEPAS system designed for methane and ethane detection is depicted in Figure 1. Two near-IR distributed feedback (DFB) butterfly-packaged laser diodes were chosen as light sources. A laser diode (LD1) emitting around 1653.7 nm with an optical power of 12 mW (AOI DFB-1653-BF-12-CW-F2-H2-N127, AOI, Sugar Land, TX, USA) was chosen to target a merged methane triplet (P1) centered at 6046.94 cm^−1^ with an overall cross-section of ≈1 × 10^−20^ cm^2^/mol at atmospheric pressure [25]. A laser diode (LD2) emitting around 1684 nm with an optical power of 8.5 mW (Eblana EP1684-0-DM-B06-FA, Eblana Photonics Ltd., Dublin, Ireland) targeted: (i) a six line merging structure (P2) of methane with an overall cross-section of 7 × 10^−21^ cm^2^/mol at atmospheric pressure located at 5938.12 cm^−1^ [25]; (ii) several ethane absorption bands related to the overtones transition of the C-H stretching [26,27], with the strongest peak (P3) being at 5937.3 cm^−1^ [28,29]. Two compact laser diode drivers (Thorlabs CLD1015, Thorlabs, Newton, NJ, USA) controlled both the temperature and the laser sources’ current. A custom-made Thorlabs fiber combiner (Thorlabs Canada ULC, Boulevard Montpellier Saint-Laurent, QC, Canada) was employed to couple the beams of the two lasers. Transmission efficiency of more than 92% was measured for both channels of the fiber coupler. The combiner was connected to the fiber port mounted on the ADM (see Figure 1), and the output beam was collimated through the tubes of the spectrophone composed of a custom T-shaped QTF having resonance frequency f_0_ of 12458.7 Hz and a dual-tube resonator system consisting of two 12.4 mm long tubes with an inner diameter of 1.6 mm. This spectrophone configuration provides both the highest signal to noise ratio enhancement with respect to the bare QTF (60×) [30] and allows an easy alignment of the laser beam through the QTF prongs spaced 0.8 mm apart. An optical power meter was employed for laser beam alignment purposes. About 99% of the incident radiation was measured passing through the spectrophone. The piezocurrent generated by the QTF was transduced into a voltage signal by means of a transimpedance amplifier. The 2f-detection wavelength modulation (WM) QEPAS technique was used by modulating the laser current with a frequency of f_0_/2 and acquiring the f_0_-oscillating component of the spectrophone signal output. The analog outputs of a NI PCIe-6363 DAQ card (National Instruments, Austin, TX, USA) were used to provide the f_0_/2 modulation to the laser drivers (outputs AO1 and AO2) and the reference signal to a Perkin Elmer 7265 (Perkin Elmer, Waltham, MA, USA) lock-in amplifier (output AO0). The QTF transduced signal was demodulated with an integration time set to 100 ms for all the measurements, if not stated otherwise. The signal was then acquired using the DAQ card, with an acquisition time of 300 ms. With the aim of developing a sensor suitable for real-time monitoring, measurements were performed by shining both lasers simultaneously through the ADM, to avoid any warm-up and temperature stability-related downtime. A LabVIEW (National Instruments, Austin, TX, USA) subroutine was developed to drive the two diode lasers and allow C1-C2 sequential monitoring. During a first 250 s-long time window (W1), the LD1 wavelength was modulated at f_0_/2 (sine waveform) and scanned across its full dynamic range (sawtooth waveform), from the threshold current of 30 mA to the maximum current of 160 mA, with a frequency of 4 mHz, while no current modulation was enabled on AO2. Once the LD1 sweep in time window W1 was complete, AO1 modulation was disabled and the LD2 wavelength was swept in the following 250 s-long time window W2, by modulating the injection current at f_0_/2 and scanning it from the threshold current of 20 mA to the maximum current of 120 mA, with a frequency of 4 mHz.

The gas handling system was realized as follows: 1% C1:N_2_, 1% C2:N_2_ and pure N_2_ cylinders (Nippon Gases Italia, Modugno, Italy) were connected to an MCQ Instruments Gas Blender GB-103 (MCQ Instruments, Rome, Italy) to produce the desired gas sample mixtures. The gas sample passed through an MKS Type 649 (MKS Instruments Inc., Andover, MA, USA) pressure controller/flow meter, the ADM, a needle valve, and finally, a vacuum pump. A pressure controller, a needle valve and a pump allowed fixing and monitoring both gas pressure and flow inside the ADM.

## 3. Results and Discussion

**Figure 2:**
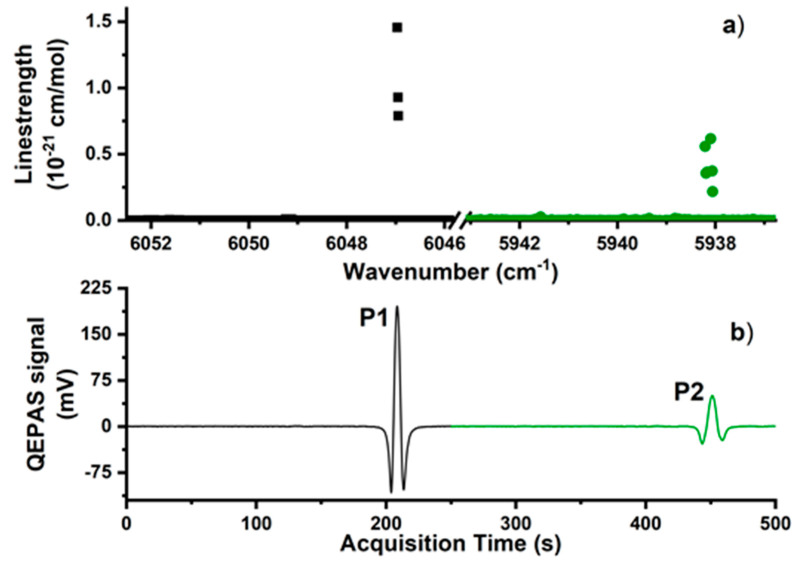
(**a**) Linestrength of methane absorption lines in the spectral ranges swept by LD1 (black dots) and LD2 (green dots). (**b**) 2f-QEPAS signal for a mixture containing 0.1% methane and 99.9% nitrogen obtained by scanning along the full dynamic range of LD1 (black curve) and LD2 (green curve). The QEPAS signals of the C1 peaks in LD1 range and LD2 range were 196.25 and 50.49 mV, respectively.

All the experimental investigation and analysis presented in this work refer to spectra acquired in both time windows, W1 and W2. The TEC temperature was set to 25 °C for LD1 and 16 °C for LD2. The multiple C1 transitions composing P1 and P2 and simulated using the HITRAN database in Figure 2a, merge at 760 Torr in the W1-W2 QEPAS spectrum shown in Figure 2b, consisting of two well-separated second derivatives of a Lorentzian profile. The methane QEPAS signals displayed in Figure 2b were acquired by flushing a 0.1% methane–99.9% nitrogen mixture through the sensor and employing a laser modulation depth of 110 mVpp for LD1 and 150 mVpp for LD2, which were identified as the values maximizing C1 signals.

The P1 and P2 peak values were measured to be 196.25 and 50.49 mV, respectively. The noise level was calculated as the standard deviation of the acquired QEPAS signal, while pure nitrogen was flushing trough the ADM. Noise levels of 145 and 150 µV were respectively measured in the LD1 and LD2 ranges. The main contributions to the noise levels are represented by (i) the thermal noise of the QTF; (ii) the electrical noise of the whole system; (iii) the photothermal signal arising from the laser beam tails hitting the spectrophone; and (iv) slow oscillations of mechanical components. For a 0.1% C1 in N_2_ mixture, no background absorption with respect the ground noise recorded in pure N_2_ was detected in LD1 and LD2 laser ranges.

**Figure 3:**
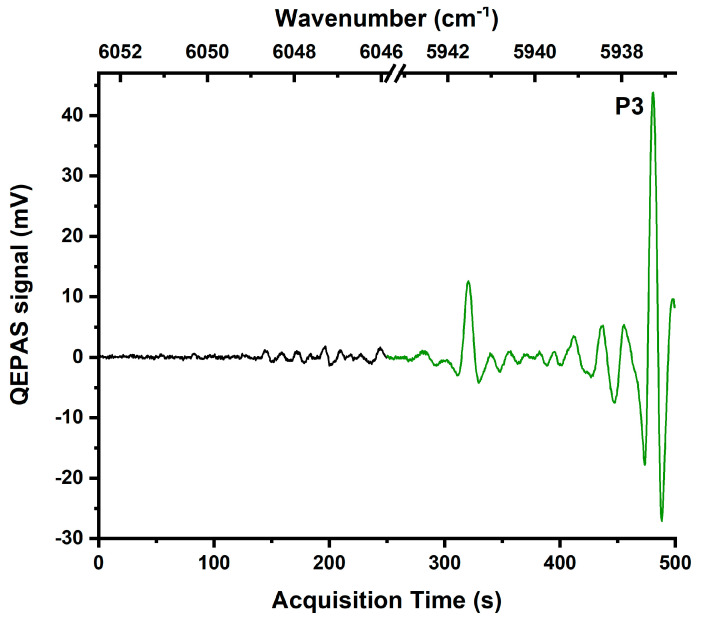
2f-QEPAS signal for a mixture containing 1% ethane and 99% nitrogen. The black curve was obtained by sweeping the full dynamic range of LD1, and the green curve was obtained by sweeping the full dynamic range of LD2.

Ethane QEPAS spectrum in Figure 3 was obtained by flushing a 1% ethane–99% nitrogen gas mixture through the ADM.

The tuning range covered by the two DFB lasers allowed targeting several ethane absorption features. The QEPAS spectrum acquired in the LD1 range can be divided into two distinguishable regions. The first one from 0 to 140 s (6052.5–6049 cm^−1^) is characterized by a standard deviation comparable to the noise floor. A standard deviation of 650 µV characterizes the second one from 140 to 250 s (6049–6046 cm^−1^) with a non-resolved absorption background. Much stronger QEPAS signals are observed in the spectral range covered by LD2, with the strongest peak (P3) falling at 480 s (5937.3 cm^−1^).

**Figure 4:**
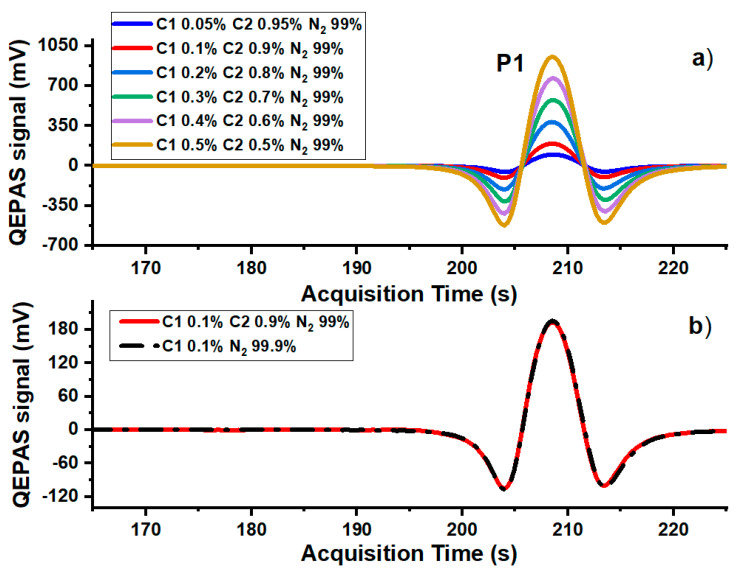
(**a**) P1 QEPAS signals measured in the LD1 emission range nearby P1 for six mixtures of C1, C2 and N_2_. (**b**) Comparison of LD1 0.1% methane QEPAS signals between mixtures containing 99.9% nitrogen, and 99% nitrogen and 0.9% ethane.

**Figure 5:**
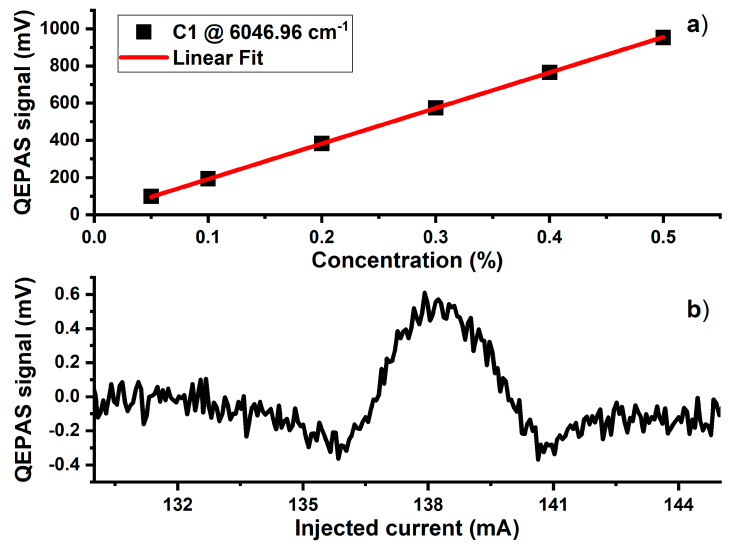
(**a**) Linearity of the 2f methane peak signal in the concentration range 0.05–0.5% in mixtures containing both C2 and N_2_. The uncertainty of the measured data points lies within the size of the depicted data point symbols. (**b**) 2f C1 QEPAS signal in the LD1 range measured for a standard-air mixture containing 1.9 ppm of methane.

**Figure 6:**
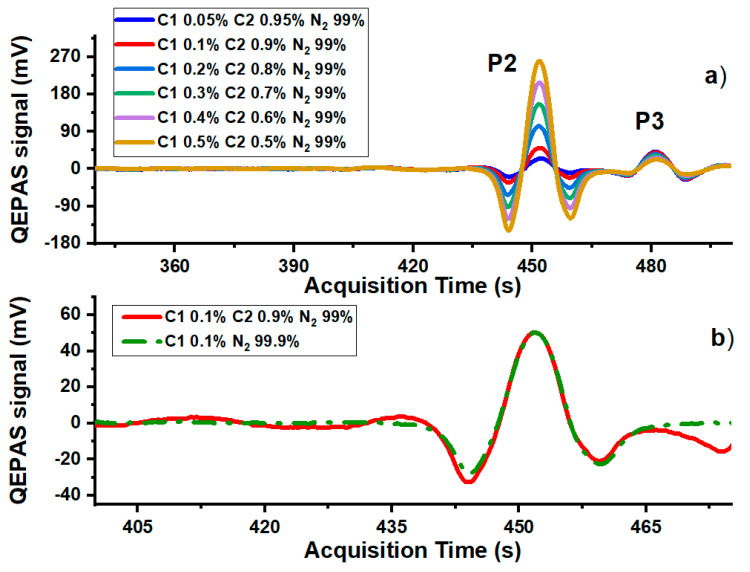
(**a**) 2f-signals in the LD2 range measured for six mixtures of C1, C2 and N2. (**b**) Comparison of 0.1% of LD2 methane QEPAS signals between mixtures containing 99.9% nitrogen, and 99% nitrogen and 0.9% of ethane.

**Figure 7:**
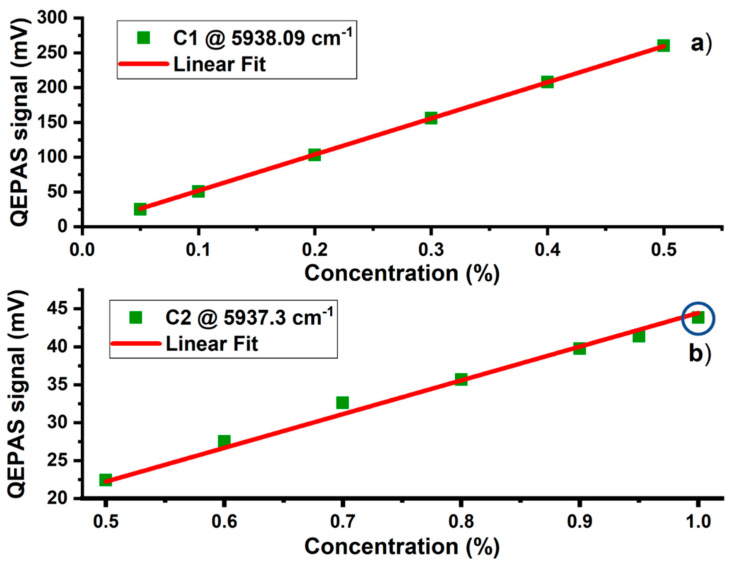
(**a**) Linearity of the 2f methane peak signal in the concentration range 0.05–0.5% in mixtures with C2 and N_2_. (**b**) Linearity of the 2f ethane peak signal in the concentration range 0.5–1% in mixtures with C1 and N_2_. The uncertainty of the measured data points lies within the size of the depicted data point symbols.

The sensor performances for methane and ethane detection in mixtures are displayed in Figure 4, Figure 5, Figure 6 and Figure 7. In Figure 4a a portion of the W1 2f-QEPAS spectra acquired for six different mixtures, simulating a 1% contamination of a pure nitrogen matrix with different C1–C2 combined concentrations, are shown.

Figure 4b shows the comparison between the P1 QEPAS spectra measured for a mixture of 0.1% of C1 in a matrix of pure N_2_, and a mixture with a C1 concentration fixed at 0.1%, while C2 concentration is at 0.9% and the rest is N_2_. It is clearly visible that the presence of 0.9% of C2 does not influence the intensity or the shape of P1, neither in terms of absorption interference nor in terms of energy relaxation as a collisional promoter. This experimental evidence is confirmed by the perfect linearity of the peak value of P1 versus the C1 concentration (Figure 5a) extracted from the 2f-QEPAS spectra shown in Figure 4a.

The slope of the calibration curve, obtained by interpolating the data in Figure 5a with a linear fit, is 1910 mV/%. The minimum detection limit (MDL) that can be reached at 100 ms of integration time is 0.76 ppm, more than two times lower than the natural C1 concentration in standard air (~1.9 ppm). This sensitivity, together with the absence of interference effects from ethane, justify the use of LD1 for continuous monitoring of the environmental CH_4_ concentration. The measurement of ambient methane in a standard air matrix is shown in Figure 5b. In order to enhance the natural C1 QEPAS peak for this measurement, an integration time of 2 s and an acquisition time of 6 s were chosen.

The QEPAS spectrum shown in Figure 5b was obtained at atmospheric pressure. Compared to the expected value of ~0.36 mV calculated from the pure N_2_ matrix calibration shown in Figure 5a, the ~0.5 mV peak value measured in standard air was slightly higher. This could be related to the effects of water vapor and O_2_ in the air matrix on methane relaxation dynamics. Acoustic wave generation relies on the transfer rate at which vibrational energy of the excited target gas molecules is converted into the kinetic energy of the surrounding molecules (V–T relaxation). This process is characterized by a relaxation time τ, which depends on the composition of the mixture according to the formula [31]:

1/τ_M_ = ∑ C_i_ ∙1/τ_M-Mi_, (1)

where 1/τ_M_ is the relaxation rate of an excited state of a molecule M, 1/τ_M-Mi_ is the relaxation rate corresponding to collisions with the i-th molecule in the gas mixture and C_i_ is the concentration of the i-th molecule. The radiation-to-sound conversion efficiency of a molecule depends on the product 2πfτ, where f is the frequency of the generated acoustic wave (f = f_0_ in our case). If this product is much lower than 1 while each component’s concentration varies, the mixture composition does not affect the QEPAS signal of the target molecule [31]. Nitrogen, water vapor and oxygen are the main air components. Methane QEPAS signal was proven to depend on water vapor concentration variations when the absolute humidity varied in the range of 1.2–1.6% [32,33], when targeting absorption transition lines in the mid-IR wavelength range at 3.3 µm. This experimental evidence indicates that a C1 QEPAS signal compensation with respect to the mixture’s absolute humidity is required.

In the near-IR, the configurations of the energy levels and the relaxation rates of each energy transfer were investigated in many different studies [31,34]. For the transitions involved in the experiment presented here, at a working pressure of 760 Torr, the relaxation rates of the excited C1 energy levels through the collisional partners are listed in Table 1:

Considering a sample of standard air composed of 1.9 ppm of methane, 20.9% oxygen, 1.86% water and the remaining part of nitrogen, the relaxation rate of C1 is in the 10^7^ s^−1^ order of magnitude. The order of magnitude of the product 2πfτ is in the 10^−3^ scale. The dominant contribution on the CH_4_ relaxation rate in standard air is the V–T relaxation of methane on water vapor. Even if the concentrations of the other components change, the methane relaxation rate does not change. Calculations using Equation (1) indicate that the water vapor influence on the methane relaxation rate saturates at H_2_O concentrations over 0.6%. Thereby, in the typical water vapor concentration range of standard air (between 0.6% and 3% [25]), no variations of the C1 QEPAS signal are expected when targeting transitions at 1.654 µm. This implies that the radiation-to-sound conversion efficiency will not change. Therefore, the methane QEPAS signal in this wavelength range at atmospheric pressure is not expected depending on water vapor or oxygen variations around the atmospheric concentration or on other components in standard air. All these assumptions avoid the necessity of C1 signal compensation.

While LD1 can guide the drone along the concentration gradients, LD2 can be used to measure both C1 and C2 in an air sample once the pollution source has been identified, to characterize the source through the C2/C1 ratio.

Figure 6a shows the QEPAS spectra in a portion of the LD2 dynamic range for the same mixtures investigated in the LD1 range (see Figure 4a).

P2 and P3 features are well-resolved in the 2f-QEPAS scans even for unbalanced mixtures (e.g., 0.05% C1—0.95% C2—99% N_2_). Additionally, in W2 the C1 exhibits no absorption background, but the C2 broadband absorption slightly influences the P2 peak profile. Indeed, as shown in Figure 6b, a small difference between the spectra related to the 0.1% C1—99.9% N_2_ (green dash dots) and 0.1% C1—0.9% C2—99% N_2_ (red line) mixtures can be appreciated just for the negative lobes. This means that the C1 concentration measurements extracted from the peak values are not affected even in a mixture with a C2 concentration almost one order of magnitude larger than C1, which is still an unlikely situation in real life.

Despite different ethane concentrations in the mixtures, the calibration with respect to P2 reflects a perfect linearity between the measured peak signal and the C1 concentration (Figure 7a).

This further experimental evidence confirms that ethane does not influence the methane QEPAS signal in this wavelength range. The slope obtained from the linear fit was 520 mV/%. The sensitivity reached at 100 ms integration time was 2.9 ppm. The lower sensitivity of P2 with respect to P1 extends the overall detectable methane concentration range to higher values, without signal saturation. In this configuration higher C1 concentrations can be detected with LD2 in the immediate vicinity of the pollution source/leakage. Combining both LD1 (W1) and LD2 (W2), this QEPAS sensor can detect methane concentrations ranging from ppb to few percent.

The calibration of the 5937.3 cm^−1^ ethane peak P3 is shown in Figure 7b. The 1% ethane peak value, highlighted with a blue circle in Figure 7b, corresponds to the peak value of Figure 3, without methane in the mixture. The linearity shown in Figure 7b demonstrates that presence of methane does not influence the ethane QEPAS signal in the mixture. The slope retrieved from the linear fit of the C2 calibration is 44 mV/%. Considering the 150 µV noise level, the ethane sensitivity at 100 ms of integration time is 34 ppm.

## 4. Conclusions

In this work, the realization of a novel, ultra-compact, near-IR QEPAS sensor aimed at UAV-assisted environmental monitoring of methane and ethane, exploiting a novel fiber-coupled ADM and a custom fiber combiner, is reported. The system employs two laser diodes in the near-IR range (AOI diode laser operating at 1653.7 nm and Eblana diode laser operating at 1684 nm). The use of the two diodes to target the overtone absorption of the C–H bond stretching at ~1.6 µm for both C1 and C2 allowed us to both keep the power consumption relatively low and reduce the overall weight of the sensor. The custom tuning fork T-QTF combined with acoustic resonator tubes allowed us to reach sensitivity levels of 0.76 ppm for methane and 34 ppm for ethane. From the perspective of implementing this sensor on UAVs, a feasible detection scheme would rely on the continuous monitoring of P1 signal to recognize changes in atmospheric methane, identify positive concentration gradients and drive the vehicles along the direction of the highest concentration slopes. Once the leak source is identified, the second detection sequence monitoring P2–P3 can be activated and the C2/C1 concentration ratios can be measured. For C1 and C2 monitoring a line-locking configuration would be also implemented, instead of full spectral scan acquisitions. Integration times of a few seconds can be set to further improve the detection limits. With an integration time of 2 s (acquisition time of 6 s) the atmospheric C1 concentration is clearly detectable. A custom board capable of providing the current modulations and processing the QTF signal will be further engineered by implementing a lock-in software system. This will reduce both the noise level due to the transimpedance amplifier integration on board and the physical size of the whole sensor. From the perspective of an UAV swarm-coordinated flight, environmental methane monitoring would allow the pollution source to be first localized by a single drone unit. A request would then be issued to all other vehicles in the swarm to aid the first unit in pinpointing more precisely the location of the leak, determining the source characteristics and triggering a repair and/or damage containment intervention in pipelines, guided by machine learning methods. The realization of an integrated optic beam delivery could further reduce QEPAS sensor size and the effects of mechanical instabilities due to the UAVs motion [35].

## Figures and Tables

**Table 1:** V–T relaxation rates of the n^th^ *ν_4_* CH_4_* excited vibrational state with the main collisional partners in standard air at a working pressure of 1 atm.

Reaction	1/τ_M-Mi_ (s^−1^)	Reference
CH_4_*(n*ν_4_*) + CH_4_ → CH_4_*[(n−1)*ν_4_*] + CH_4_	8 × 10^5^	[31]
CH_4_*(n*ν_4_*) + N_2_ → CH_4_*[(n−1)*ν_4_*] + N_2_	8 × 10^4^	[31]
CH_4_*(n*ν_4_*) + O_2_ → CH_4_*[(n−1)*ν_4_*] + O_2_	1.3 × 10^5^	[31]
CH_4_*(n*ν_4_*) + H_2_O → CH_4_*[(n−1)*ν_4_*] + H_2_O	8.2 × 10^7^	[34]

## Abbreviations

The following abbreviations are used in this manuscript

ADM	Acoustic Detection Module
C1	Methane
C2	Ethane
LD1	Laser Diode 1
LD2	Laser Diode 2
Near-IR	Near-InFrared
QEPAS	Quartz Enhanced PhotoAcoustic Spectroscopy
QTF	Quartz Tuning Fork
T-QTF	T-shaped QTF
UAV	Unmanned Aerial Vehicle
